# DNA as a Double-Coding Device for Information Conversion and Organization of a Self-Referential Unity

**DOI:** 10.3390/dna4040032

**Published:** 2024-11-19

**Authors:** Georgi Muskhelishvili, William Nasser, Sylvie Reverchon, Andrew Travers

**Affiliations:** 1 School of Natural Sciences and Biotechnology, Agricultural University of Georgia, 0159 Tbilisi, Georgia; 2 INSA-Lyon, CNRS, UMR5240, Microbiologie, Adaptation, Pathogénie, Université Lyon 1, F-69622 Villeurbanne, France; 3 MRC Laboratory of Molecular Biology, Francis Crick Avenue, Cambridge Biomedical Campus, Cambridge CB2 0QH, UK

**Keywords:** self-referential system, DNA information, supercoiling, gradients, nucleoprotein complexes, inter-conversion of logically distinct information types

## Abstract

Living systems are capable on the one hand of eliciting a coordinated response to changing environments (also known as adaptation), and on the other hand, they are capable of reproducing themselves. Notably, adaptation to environmental change requires the monitoring of the surroundings, while reproduction requires monitoring oneself. These two tasks appear separate and make use of different sources of information. Yet, both the process of adaptation as well as that of reproduction are inextricably coupled to alterations in genomic DNA expression, while a cell behaves as an indivisible unity in which apparently independent processes and mechanisms are both integrated and coordinated. We argue that at the most basic level, this integration is enabled by the unique property of the DNA to act as a double coding device harboring two logically distinct types of information. We review biological systems of different complexities and infer that the inter-conversion of these two distinct types of DNA information represents a fundamental self-referential device underlying both systemic integration and coordinated adaptive responses.

## 1 Introduction

**Figure 1:**
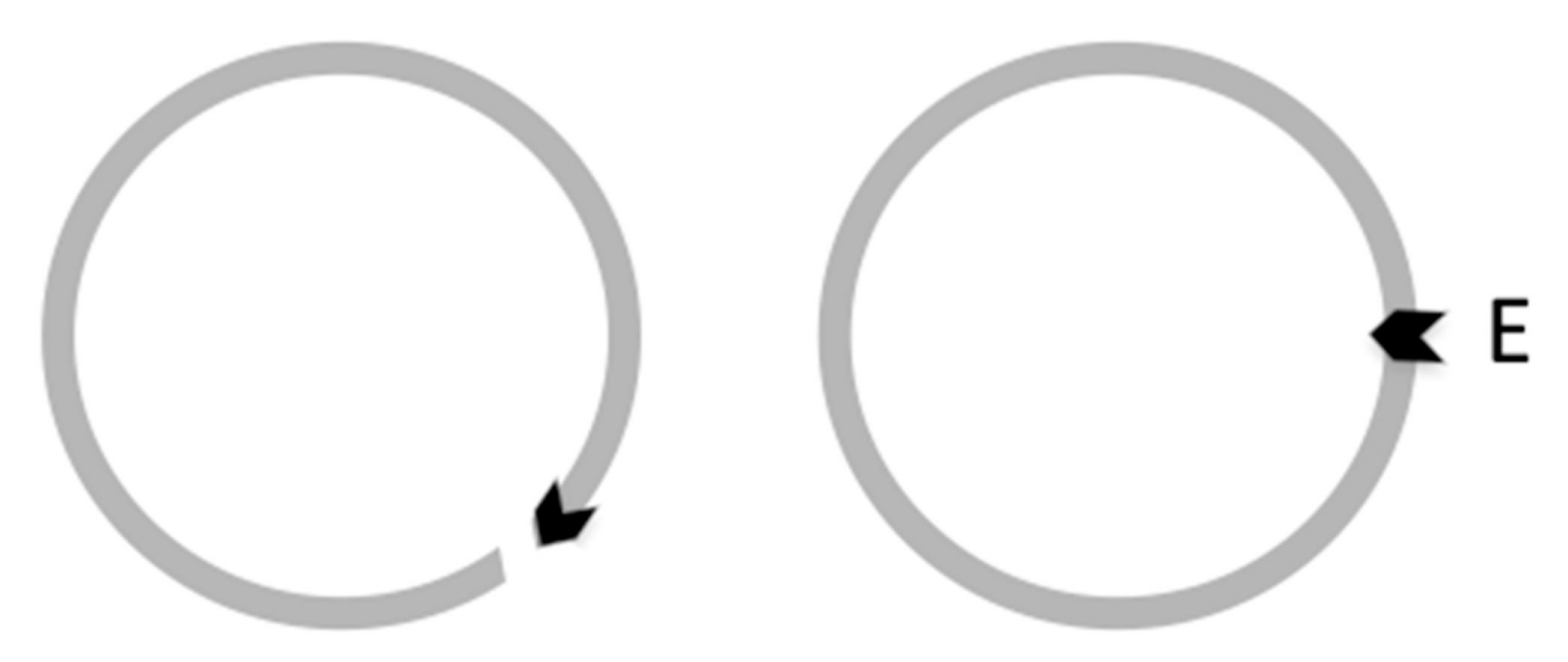
The self-referential organization of living system is represented by an arrow, which closes on itself (**left panel**). The self-pointing arrow is a symbol for the condition in which the system divides itself into that which monitors and that which is monitored [1]. The system thus constitutes an isolated, operationally closed circuit. The environment (the space outside of the circle circumference) is marked as ‘E’ (**right panel**). The environmental impact on the system is indicated by the black arrow crossing the circle circumference from outside to inside. Unless this environmental impact is deteriorating, the operational closure of the system is retained.

The distinctive organizational hallmark of living systems is their ability to self-reproduce. For that matter, living systems are regarded as ‘autopoietic’ self-referential systems implying the capacity to monitor oneself, that is, to perpetually assess their status quo [1,2]. At the same time, living systems are capable of monitoring their surroundings and eliciting a functionally coordinated adaptive response to environmental change (Figure 1). The capacity of ‘monitoring oneself’ assumes that the system divides itself, as it were, into two parts, that which monitors and that which is monitored. Furthermore, monitoring oneself and monitoring the environment appear as separate tasks utilizing different sources of information. In multicellular eukaryotes, the relative independence of the information used to control cellular reproduction and functional specialization is apparent in the separation of the processes of proliferation and differentiation [3] observed in most cells, especially during development.

In unicellular organisms such as bacteria, alteration of, e.g., the cell motility in response to environmental signals (chemotaxis) and the process of cell division are also regulated independently. For example, the control of bacterial cell density (quorum sensing) is executed by autocrine/paracrine signaling pathways involving autoinducer molecules [4], whereas bacterial chemotaxis is induced by environmental factors including, e.g., those produced by plants [5–7]. In the *Caulobacter* system, cell differentiation can be decoupled from DNA replication [8], and the processes of cell division and differentiation are distributed between the two morphologically and developmentally distinct daughter cells [9].

However, both in eukaryotes and prokaryotes, the information underpinning the apparently independent processes of reproduction and adaptation is encoded in the very same DNA genome being reflected in, and largely governed by, the genetic control mechanisms. Furthermore, even if cellular reproduction and the adaptive environmental response may utilize different information sources and independent regulation mechanisms, it is obvious that the intrinsic organization of a cell endows it with the capacity to behave as a whole, indivisible unity in which apparently independent processes and mechanisms are both coordinated and integrated [3,7,10]. Importantly, a coordinated switch in gene expression during the transition between bacterial motile and biofilm lifestyles appears to involve a change in chromosome structure [11]. Additionally, the switching between alternative gene expression programs both during the growth cycle and in response to various stress impacts involves coordinated alterations of DNA topology, coherently modulating the gene expression in extended chromosomal domains [12,13]. So then, assuming that both the genetic expression and the structural dynamics of the genomic DNA polymer are intimately involved in this integration process, the central question to address is the nature of the coordinating device.

## 2 DNA Is a Source of Two Distinct Types of Information

**Figure 2:**
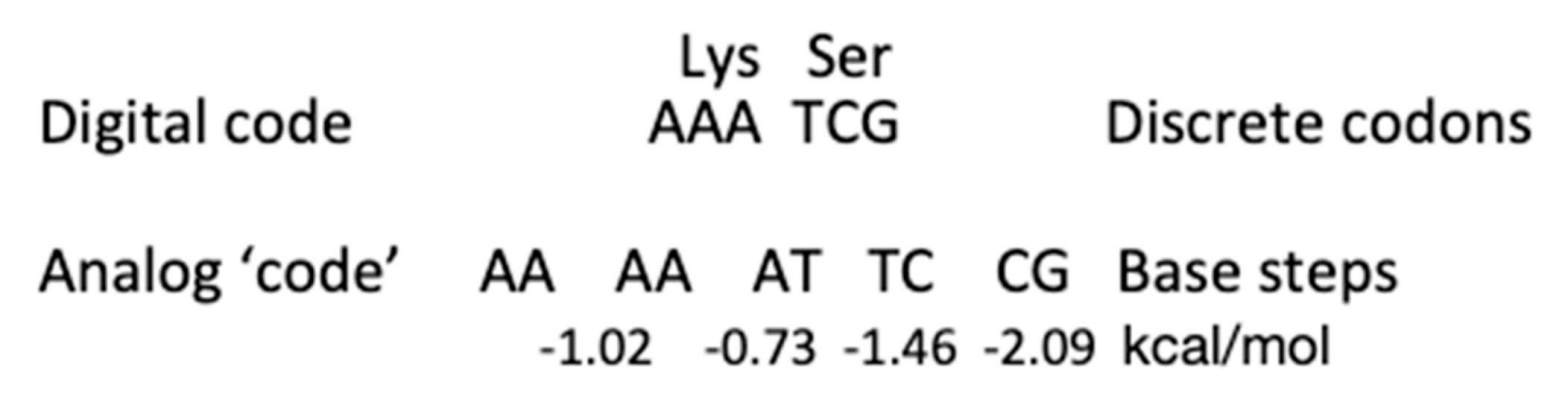
Relation between the digital genetic code and the analog information stored in overlapping base steps of the DNA. Two deliberately chosen triplets coding, e.g., for lysine and serine are indicated as ‘digital code’. Indicated below as analog ‘code’ are the five consecutive overlapping base steps harbored in these two codons. The free stacking/melting energies of the base steps [14] are indicated underneath (see also Table 1).

The still largely underappreciated characteristic of the double helical DNA polymer is that it is a source of two logically distinct types of information. One is the well-known linear genetic code, which is discontinuous (digital), being embodied in discrete triplets of base pairs—the codons (Figure 2). The other source of information stored in the DNA is of a continuous (analog) nature, being embodied in the juxtaposition of distinct base steps, which partly overlap [14–18].

**Figure 3:**
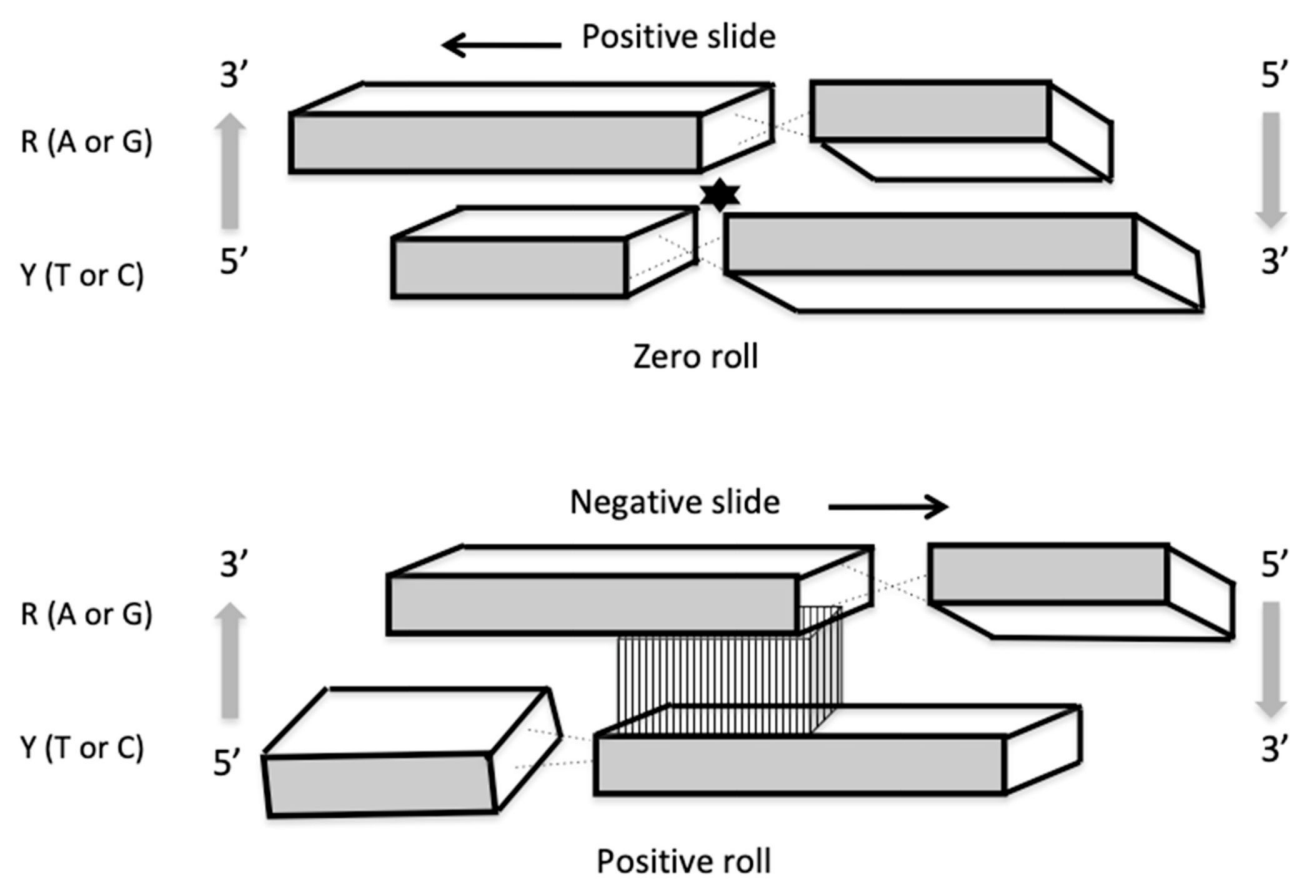
Pyrimidine−purine (YR) base steps are flexible and can adopt various conformations. Purine and pyrimidine bases are indicated by large and small rectangles, respectively. The asterisk in the upper panel indicates the potential steric clash between large purine bases, which is avoided by the positive slide of the base pairs. In the lower panel, this base step has a negative slide and positive roll, and this alternative configuration is stabilized by increased cross-chain stacking interactions between the large purine bases (vertically striated region between the large rectangles). The minor groove side of the bases is shaded (after [18]).

Since in contrast to the codons, the base steps are overlapping (that is, each first base of any base step is the second base of a previous step and each second base of any base step is the first base of a following step), it is exactly this latter feature, which confers the characteristic of continuity to the DNA analog ‘code’. Importantly, various base steps are characterized by distinct stacking/melting energy levels (Table 1) and can also adopt different preferential conformations [15–18] (Figure 3).

The contiguous base steps favoring various local conformations can determine the 3D configuration and average trajectory of the DNA [15,16,18,19]. Importantly, the base stacking and, accordingly, the conformation of DNA base steps, can be modulated by environmental conditions inducing alterations in the DNA twist eventually affecting the DNA helical repeat and the torque accommodated by the double helix. The configuration of DNA depends both on the sequence organization and average superhelical density [20–22], as well as on the size of the affected topological domain [23].

In the bacterium *E. coli*, the DNA superhelical density varies as a function of cellular energy charge, which depends on, and changes with, the environmental conditions. The level of negative DNA superhelicity varies as a function of the ATP/ADP ratio, primarily because ATP is utilized by DNA gyrase, an enzyme introducing negative supercoils into the DNA [24–27]. Both the ATP/ADP ratio as well as the gyrase activity increase on nutritional shift-up, e.g., when the starved bacterial cells are inoculated in fresh growth medium. However, at this stage, there is also a more direct effect on the DNA topology (namely, on the DNA twist) mediated by the changing ionic composition [28–33]. In other words, changing environmental conditions altering the DNA superhelicity eventually stabilize distinct DNA structures depending on the sequence organization and size of the affected domain. Ultimately, the sensing of available metabolic energy and ionic composition by DNA would select the configuration and topology optimally adapted to a given environmental impact. In turn, alterations in the DNA configuration are relevant to gene expression, as the DNA binding ligands, architectural proteins, and enzymes (e.g., the transcription and replication machinery) show preferences for a particular DNA topology [34–44]. The DNA binding proteins can in turn stabilize different DNA deformations such as bending, over- or undertwisting, wrapping, looping, and bridging, as well as can constrain DNA supercoils [45–49]. All these effects are pertinent, as the various assembled nucleoprotein complexes modulate the genetic expression. Furthermore, when translocating along the DNA template, the transcription and replication machineries directionally modulate the DNA superhelicity, inducing positive supercoils ahead and negative supercoils in their wake [50]. Diffusion of these induced free supercoils can distinctly affect the activity of neighboring genes in the genome [51–53]. In addition, this topological differentiation of DNA on opposite sides of the moving DNA translocases has the potential to spatially organize the binding of regulatory proteins recognizing distinct DNA supercoil structures [23].

Thus, in principle, coordination of different genetic programs (e.g., those governing self-reproduction and those for adaptive responses) could be achieved simply by arranging the genomic DNA analog information in such a way as to couple the emergence of distinct 3D DNA structures and particular DNA topologies to different internal and external impacts on the one hand and, on the other hand, to employ these distinct structures for selective and coordinated readout of the digital (genetic) code optimizing the expression of traits apt for coping with the given demands. The expression of different genetic programs in response to both environmental and internal signals would then be integrated and coordinated by variation of a single, continuous tunable parameter sensitive to both internal alterations and environmental change. Conceivably, the superhelicity of DNA serving as an interface between the external and internal milieu is the most plausible contender for the role of the pivotal variable adjusting the dynamics of DNA analog information (i.e., the genomic DNA configuration) and the pattern of gene expression in response to both internal and external signals. Indeed, in bacteria, the induction of distinctly different patterns of gene transcription coupled to the activation of disparate genetic functions has been observed in response to the directional modulation of DNA superhelical density by environmental stress or topoisomerase poisons and inhibitors as well as in response to topoisomerase gene mutations [12,13,54–59].

**Figure 4:**
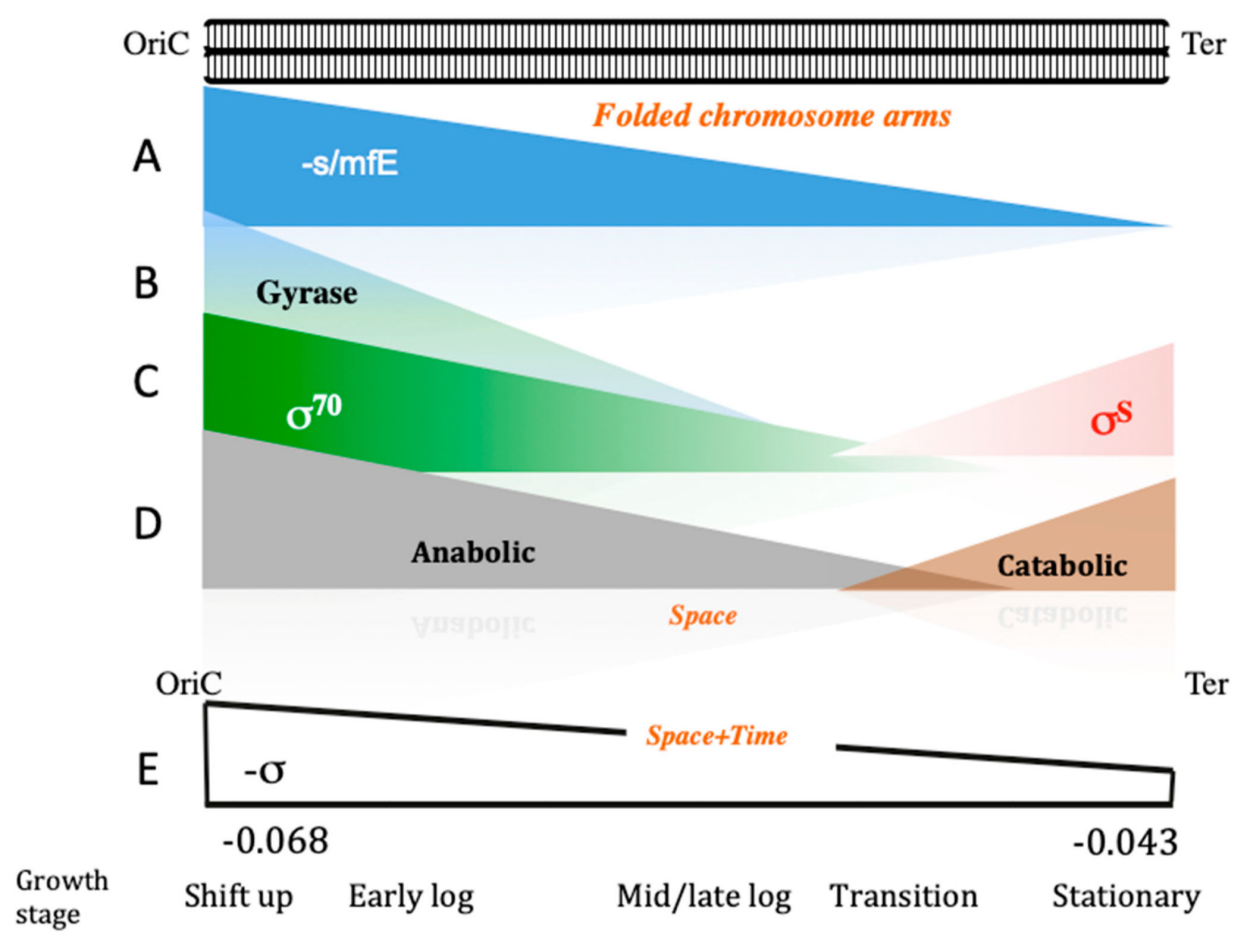
Organization of the gene expression program during the bacterial (*E. coli*) growth cycle. The circular bacterial chromosome is indicated on the top in folded form, with two arms aligned along the *oriC-t*er axis. Indicated below, all arranged along the *oriC-t*er axis are (**A**) the spatial gradient of the DNA average negative stacking/melting free energy (approx. the G/C content); (**B**) the frequency distribution of gyrase binding sites; (**C**) the frequency distribution of the σ70 and σS binding sites; **(D)** the spatial organization of anabolic and catabolic genes; (**E**) the spatiotemporal gradient of negative superhelical density (−σ), which changes both temporally with growth phase (from ∼−0.068 on shift-up to ∼−0.043 in stationary phase [56]) as well as forms a spatial gradient along the *oriC-ter* axis of the chromosome [44,66]. Thus, (**A**−**D**) show the distribution of variables in space, whereas **(E)** indicates the distribution of superhelical density both in space and in time. This spatiotemporal gradient is proposed to coordinate the expression of the anabolic and catabolic genes during the bacterial growth cycle [66,71].

In bacteria, the role of DNA topology in coordinating the genetic adaptive response with various environmental cues is well documented [13,60,61]. Additionally, in experimental evolution studies, the modulation of global regulatory networks and DNA topology were identified as the main internal factors subject to the process of selection [62,63]. During the bacterial growth cycle, the successive stages of cell reproduction (also known as the exponential growth phase) and maintenance (that is, the stationary phase) are long known to be associated with distinct—high and low, respectively—negative superhelical densities of the DNA [64]. Notably, the spatial separation of relatively G/C-rich and relatively A/T-rich sequences, organized respectively around the *oriC* and *ter* poles of the *E. coli* chromosome, allows for the temporal separation of gene expression at the two chromosomal poles due to growth phase-dependent changes in the superhelicity. Indeed, the chromosomal *oriC* pole is not only G/C-rich relative to the *ter* pole but is also enriched for gyrase binding sites [54,65,66]. On nutritional shift-up, the increase in negative superhelicity at this chromosomal pole is reinforced by the production of negative supercoils trailing both the translocating replisomes and the trains of RNA polymerase σ70 holoenzyme molecules transcribing the numerous strong ribosomal RNA operons, all of which are directionally oriented from *oriC* towards the terminus of chromosomal replication [65]. The vegetative σ70 RNA polymerase and the stationary phase σS holoenzymes respectively prefer highly supercoiled and relaxed DNA templates and, accordingly, are activated in succession during the growth cycle [34,36,67,68]. The frequency distributions of the σ70 and σS binding sites form correspondingly decreasing and increasing spatial gradients along the chromosomal *oriC-ter* axis [66]. Thus, the *oriC* pole (the Ori macrodomain and the flanking left and right non-structured domains) of the *E. coli* chromosome is enriched for σ70 binding sites and transcribed by σ70 RNA polymerase both earlier and more actively than the *ter* pole enriched for σS binding sites, giving rise to early gene products underpinning fast growth and replication [12,66,69]. Furthermore, the anabolic and catabolic genes are respectively enriched at the *oriC* and *ter* poles of the *E. coli* chromosome [70]. As a result, anabolic pathways are activated early during the reproduction stage under conditions of high negative superhelicity, and catabolic pathways are activated later under conditions of low negative superhelicity characteristic of the maintenance stage [12,56]. Thus, during the bacterial growth cycle, the temporal separation of anabolic (reproductive) and catabolic (maintenance) gene expression ‘subprograms’ is achieved by strategic spatial organization of the DNA analog information (such as the *oriC-ter* gradients of the DNA thermodynamic stability and the relative frequencies of the gyrase, σ70, and σS binding sites) coordinated with the asymmetric enrichment of anabolic and catabolic genes around the chromosomal poles (Figure 4).

**Figure 5:**
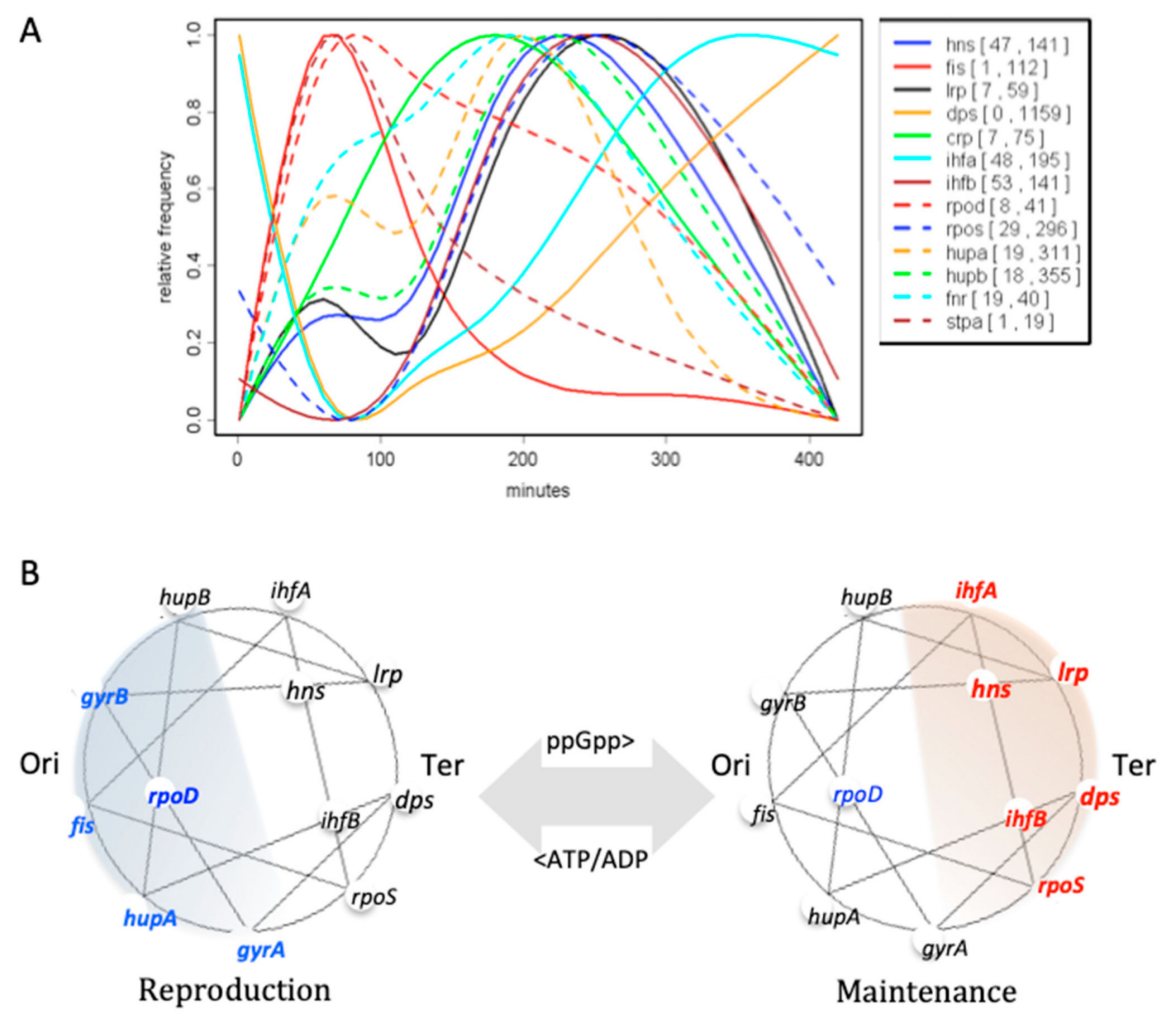
Switch between the reproduction and maintenance programs in *E. coli*. (**A**) Growth phasedependent expression of the NAP and sigma factor genes The different expression curves were normalized to [0;1] to compare them in one plot. Minimum and maximum values are indicated in brackets in the legend. Abscissa—time in minutes after inoculation of cells in fresh growth medium. The *Escherichia coli* CSH50 overnight (16 h) cultures were inoculated at an initial OD600 of 0.1 in rich double yeast-tryptone (dYT) medium and grown in a fermenter under constant pH 7.4 and high aeration (5 L air per min) at 37 °C for 7 h (420 min). Samples for RNA-seq were taken at 1, 2, 3, 5, and 7 h after inoculation. (Graph, courtesy of Patrick Sobetzko). (**B**) The circular chromosomes are depicted with the Ori and Ter poles indicated. The early regulatory genes are indicated in blue (**left panel**), and the late regulatory genes are indicated in red (**right panel**). The original position of these genes on the circular chromosome is approximated. Note that the reproduction and maintenance regulators are located around the opposite poles of the chromosome. Colored areas indicate the putative spatiotemporal concentration gradients of regulators. Connecting lines indicate the crosstalk between regulatory genes [72]. The ‘alarmone’ ppGpp produced during a shortage of nutritional resources acts as a switch from the reproduction to maintenance program. Conversely, a high ATP/ADP ratio (established, e.g., on nutritional shift-up) favors the commencement of reproduction.

In addition to the enrichment of anabolic and catabolic genes around opposite chromosomal poles, the order of cognate regulatory genes along the *oriC-ter* axis is also correlated with their successive expression during the growth cycle [66]. The genes for reproduction stage regulators are located in the vicinity of the *oriC* pole, whereas the genes for maintenance function regulators are positioned closer to the *ter* pole of the chromosome (Figure 5).

Therefore, these regulators are also expressed sequentially: first, because as already mentioned, on the commencement of growth, the *oriC* pole is activated earlier than the *ter* pole, and second, because the iterative rounds of chromosomal replication initiation at *oriC* increase the copy numbers of early regulatory genes located in its vicinity relative to that of the genes of maintenance regulators located closer to the chromosomal replication terminus [12,66,73–75]. Most important among these sequentially expressed regulators, in addition to the DNA topoisomerases and RNA polymerase sigma factors, are the highly abundant nucleoid-associated proteins (NAPs), which potentially form spatiotemporal gradients (Figure 5) interacting with cognate binding sites spatially organized in the genome [66,76–80]. Importantly, the abundant NAPs bind DNA with different affinities depending on its 3D structure and are capable of constraining supercoils and stabilizing topological domains and thus of partitioning and storing the superhelical energy [81–83], which can be used to do work, e.g., to separate the DNA strands and facilitate transcription initiation.

**Figure 6:**
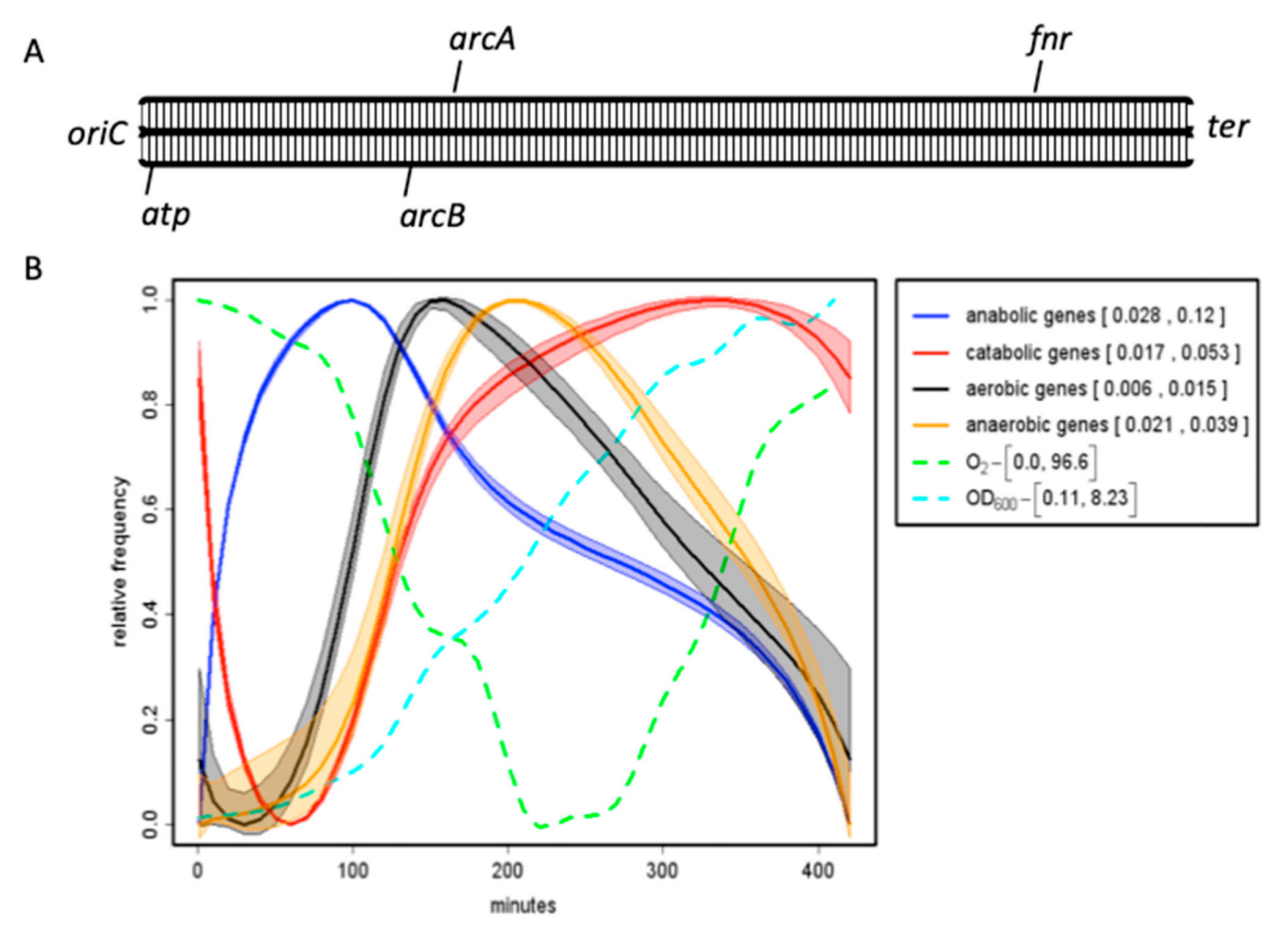
Chromosomal order of regulators and temporal pattern of regulated gene expression. (**A**) Spatial ordering of aerobic/anaerobic growth regulatory genes on the *E. coli* chromosome along the *oriC−ter* axis. Genes on the clockwise (right) replichore are indicated on the upper bar, and genes on anti-clockwise (left) replichore are indicated on the lower bar. The *atp* operon encodes ATP synthase. *arcA/arcB* encode a two-component system active under microaerobic conditions [86,87]. ArcA also represses *rpoS* encoding the stationary phase sigma factor [88]. *fnr* has a dominant role under more strictly anaerobic conditions [86]. (**B**) Temporal dynamics of expression of various gene classes. The *Escherichia coli* CSH50 overnight (16 h) cultures were inoculated at an initial OD600 of 0.1 in rich double yeast-tryptone (dYT) medium and grown in a fermenter under constant pH 7.4 and high aeration (5 L air per min) at 37 °C for 7 h. Samples for RNA-seq were taken at 1, 2, 3, 5, and 7 h after inoculation. The different curves were normalized to [0;1] to compare them in one plot. The envelopes of the curves indicate the standard deviation at 10% random remapping of the expression patterns to genes. Minimum and maximum values are indicated in brackets in the legend. Expression values (anabolic, catabolic, aerobic, anaerobic) in brackets are normalized to the expression of all genes. The optical density and partial oxygen pressure are indicated, respectively, by the dashed light blue and green lines. Note the correlation between the maximal expression of anaerobic genes and minimal partial oxygen pressure. (Graph, courtesy of Patrick Sobetzko).

Similar organizational logic applies to the operation of the aerobic/anaerobic switch during the bacterial growth cycle. The *atp* operon responsible for ATP production under aerobic growth conditions is located in close vicinity of *oriC*, while the *fnr* gene, encoding the major DNA binding regulator of anaerobic growth, is located in the vicinity of *ter*. Accordingly, the *arcA* and *arcB* genes encoding the two-component system responsible for gene regulation under conditions of microaerobiosis are located in between *atp* and *fnr* (Figure 6A). Thus, again, these regulatory genes are spatially ordered in the genome

according to their sequential requirement during growth, while the expression of aerobic and anaerobic gene groups appears to be correlated with the gradual alteration of oxygen partial pressure (Figure 6B). As with nutritional shift-up, topoisomerase activities are involved in the regulation of DNA supercoiling during aerobic−anaerobic transitions in *E. coli* [84], whereby the growth under high-oxygen conditions is correlated with the high negative superhelicity of plasmid DNA [85].

## 3 Coupling of Logically Distinct Types of Information

The pivotal question is how the coupling of DNA analog information (i.e., the spatial distribution of DNA torsional energy, which is a continuous variable) with the digital information (i.e., the selective expression of unique genes manifesting a discontinuous pattern) is accomplished in the genome. More compellingly, how do the DNA analog information and digital code communicate with each other? In bacteria, it has been shown that variations in the G/C content of the promoter sequence context [55,89] as well as the peculiar sequence organization of the different promoter elements such as, e.g., the deviation of the −35 hexamer from the consensus sequence, as well as the G/C-richness and/or extension of the discriminator sequence and the length of the spacer between the −10 and −35 hexamers confer the ability to distinctly respond to alterations in the DNA superhelical density [90–95]. Additionally, the sequences located upstream of the core promoter and characterized by anisotropic bending modulate the response to DNA superhelicity [91,96–98]. Thus, a simple way to produce a coordinated transcriptional response to the changes in supercoiling would be to put all the functionally relevant genes under the control of promoters with similar sequence organization, and indeed, that is the case for many stringently regulated genes (that is, the genes down-regulated by the alarmone ppGpp; see below) including the stable RNA (transfer and ribosomal RNA) operons [99]. However, several studies identified supercoiling-dependent, spatially extending coherent gene expression patterns in the bacterial genome that cannot be readily expounded by the promoter sequence similarity scenario [12,13,54,80,82,100]. Various explanations have been proposed for the organization of such extended topological domains including coherent domains of gene expression (also known as CODOs) [12,13,71,101], but the issue remains controversial [71,102–104]. Importantly, CODOs were found to harbor distinct genetic functions [12,13,71] consistent with the spatial coupling of the DNA analog information and the digital code in the genome.

## 4 Switching Between Alternative Gene Expression Programs

In bacteria, global alterations in gene expression can be induced not only by alterations in DNA superhelicity but also by small intracellular effectors, such as the nucleotide guanosine tetraphosphate (ppGpp). In metazoan cells, the role of ppGpp is less clear [105], while in bacteria, it serves as an alarmone, reprograming the cell physiology by interacting directly with the transcription and translation machinery [106,107]. However, ppGpp also interferes with replication initiation by modulating the DNA topology at *oriC* [108]. For that matter, ppGpp, the production of which is sharply induced during a shortage of nutritional resources, appears to act as a switch curtailing cell reproduction and promoting the establishment of the maintenance program.

This ppGpp-dependent switch in gene expression occurs on exhaustion of nutritional resources at the later stage of growth. At this stage, ribosome production and gyrase activity subside, whereas the sharply increased ppGpp concentration facilitates the compositional change in the transcription machinery, e.g., the partial substitution of σ70 by stationary phase σS factor in the RNAP holoenzyme, and, for that matter, ppGpp also switches the supercoiling preferences of the polymerase. While RNA polymerase is a direct target of ppGpp, the ppGpp sensitivity of the σ70 holoenzyme in vitro can be attenuated by increased DNA superhelicity [109]. In addition, ppGpp appears to stabilize the so-called ‘tight’ conformer of the σ70 holoenzyme at the expense of the ‘ratcheted’ conformer favoring supercoiled DNA (Malcolm Buckle, G.M. and A.T., manuscript in preparation). Furthermore, the composition of the abundant NAPs changes at this stage such that overall, the intracellular milieu and the bacterial chromatin composition facilitate the transcription of more relaxed and relatively A/T-rich DNA around the terminus of replication (the Ter macrodomain), which is enriched for genes involved in maintenance functions. The ppGpp-facilitated growth phase-dependent substitution of σ70 by the stationary phase σS factor in the RNAP holoenzyme is associated with switching between the reproduction and maintenance programs and thus resembles the genetic switch between the alternative growth pathways of temperate bacterial phages such as phage λ. The λ switch is also sensitive to both the cell density and metabolic state [110,111], as well as to the supercoiling level of the DNA [112,113].

In these two systems, despite the huge difference in complexity, there is a notable organizational similarity manifest in the conversion of distinct information types occurring during the establishment of both the bacterial switch between the reproduction and maintenance programs and the λ phage switch between the lytic and lysogenic pathways. In the latter case, the system is much simpler, and ultimately, the switch boils down to competition between two DNA binding transcriptional regulators (the Cro and CI repressors) for binding specific operator sites in the λ regulatory region. However, in both systems, first, the information of a continuous (analog) type is produced and then converted into information of a discontinuous (digital) type.

**Figure 7:**
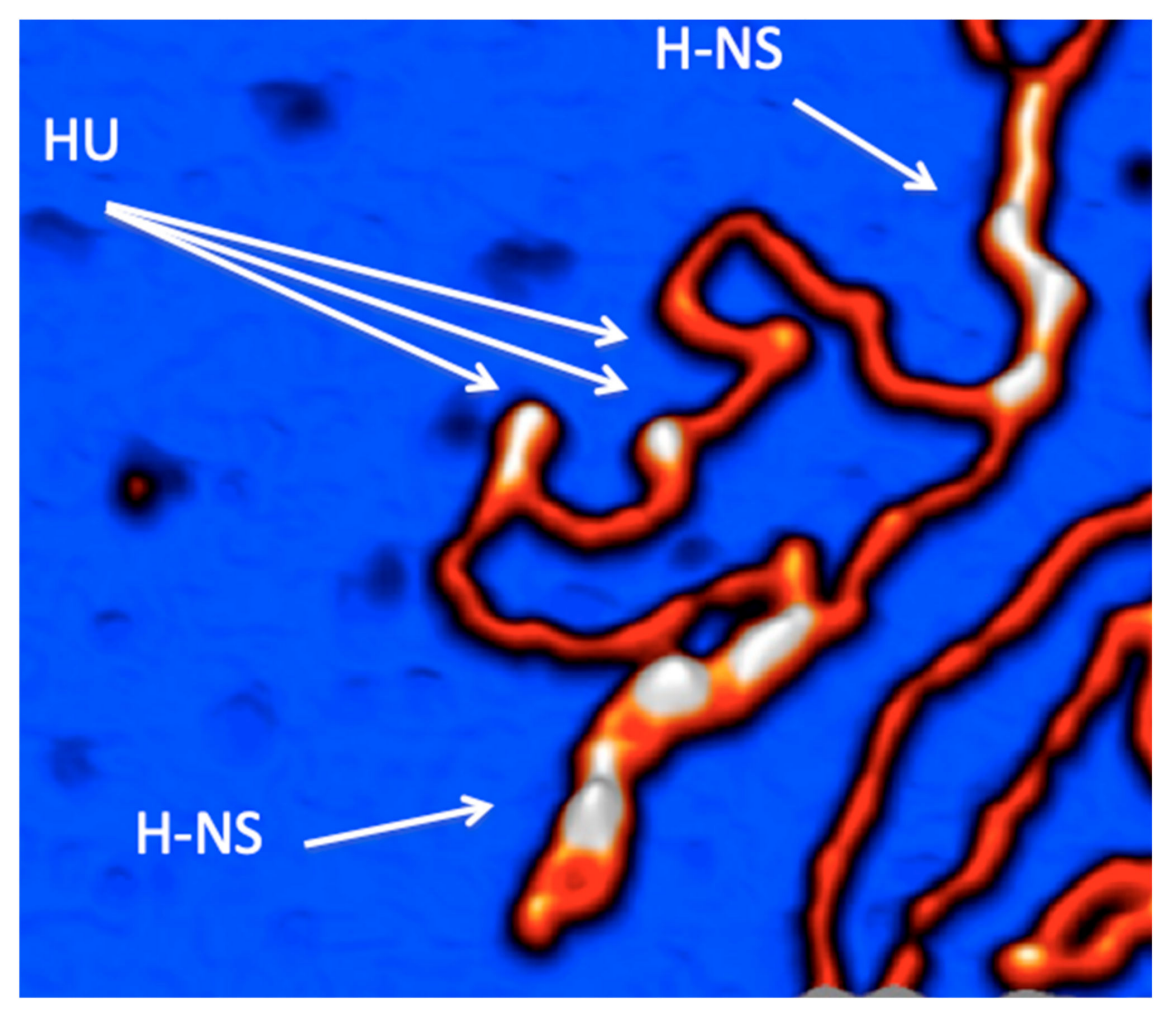
Competition between the bacterial NAPs, HU, and H-NS, which stabilize alternative supercoil structures on binding DNA. Distinct supercoil structures stabilized by HU and H-NS are indicated by white arrows. HU stabilizes more open toroidal coils, whereas H-NS stabilizes tightly interwound, stiff plectonemic DNA structures (AFM image, courtesy of Sebastian Maurer).

In the *E. coli* system, as mentioned above, analog information is manifest in the *oriC-ter* skew of DNA binding site frequencies for DNA gyrase, σ70, and σS, interacting with the changing ratio of the RNAP σ70 and σS holoenzymes, the spatiotemporal gradient of the chromosomal superhelical density, and the temporal concentration gradients evident in various growth phase-dependent levels and combinations of NAPs (Table 2). These DNA architectural proteins form distinct spatiotemporal patterns of regulatory nucleoprotein complexes in the genome [69,114–116]. The NAPs compete for the stabilization of alternative supercoil structures (Figure 7) but can also cooperate depending on the DNA sequence organization [47,117]. The mutations of NAP genes alter both the gene expression patterns and DNA topology, consistent with the notion that NAPs coordinate the growth phase-dependent chromosome structure and function [11,13,56,65,118–120].

**Figure 8:**
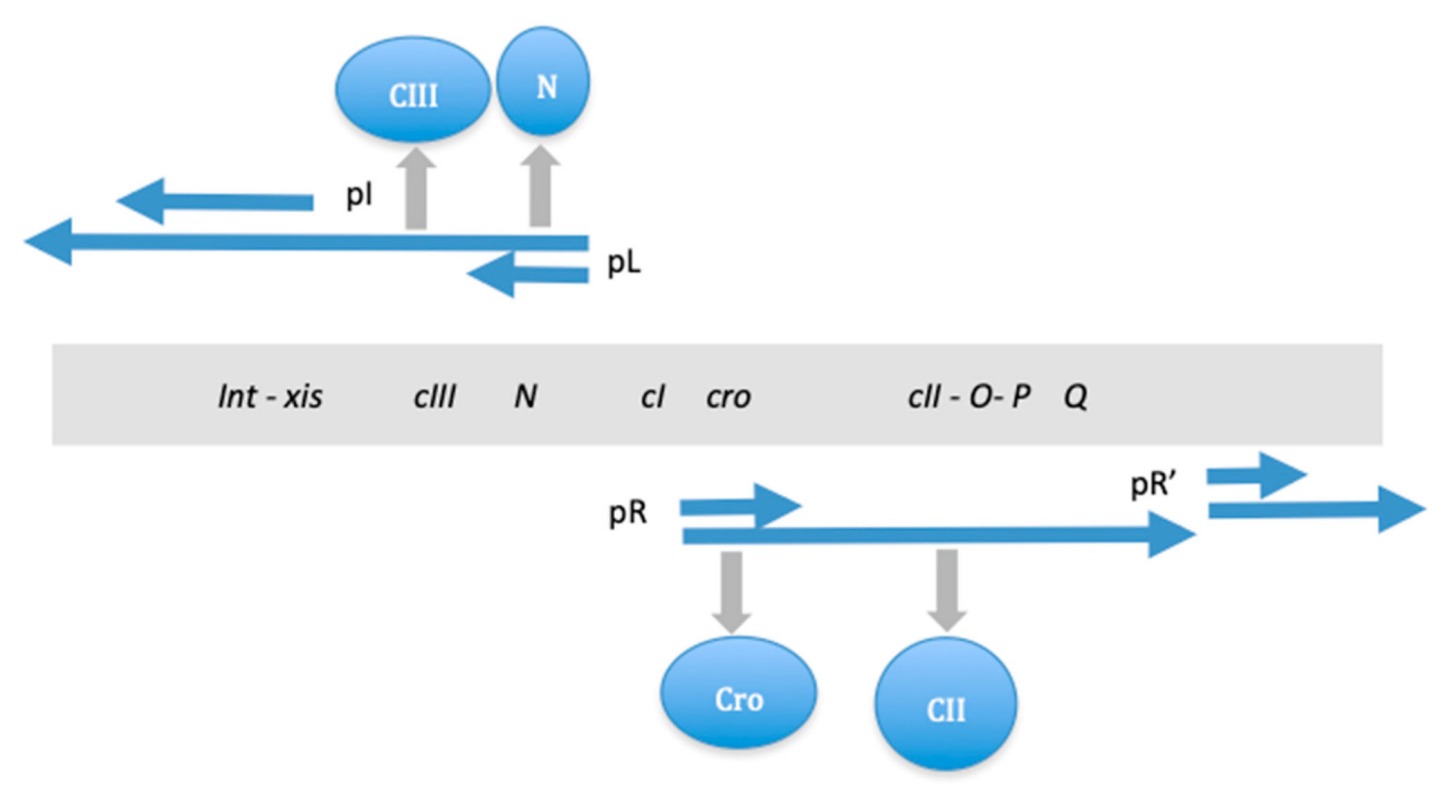
Regulation of the lysis−lysogeny decision in λ phage development. Gene and early transcription map of λ is shown (simplified). Genes are indicated in the shaded rectangle. The early transcripts produced from the pL and pR promoters are shown as blue arrows. The immediate early gene (short arrows) products are N and Cro. On extension of the transcripts known as ‘delayed early transcription’ (long arrows underneath), the CIII and CII proteins are produced. The N and Cro proteins support lytic development, whereas the CIII and CII proteins support lysogeny. Note that the synthesis of transcripts of different lengths (i.e., the generation of analog information) results in the production of distinct sets of specific proteins (digital information). O and P are DNA replication genes involved in lytic growth. Q protein turns on the late genes for the production of phage tails and heads. *cI, cII, cIII*, and *int* genes are involved in the establishment of lysogeny. The *xis* gene (together with *int*) is involved in the excision of the integrated prophage. Cro and CI are repressor proteins competing for binding at the operator sites in the λ regulatory region. Critical for the active production of CI repressor is the CII protein, the stability of which in turn is sensitive to physiological conditions.

In the case of bacteriophage λ, the analog information is manifest as the continual bidirectional extension of transcription initiated from the divergent pR and pL promoters located in the λ control region, producing on extension distinct sets of regulatory proteins, including those involved in sensing physiological conditions (e.g., CII) and eventually, by modulating the CI/Cro repressor ratio, favoring either the lytic or lysogenic pathway (Figure 8). Continual transcription is both contingent on and also results in the formation of a spatiotemporal pattern of regulatory nucleoprotein complexes in the λ genome.

In *E. coli*, the switch between the reproduction and maintenance stages is primarily dictated by the energy status, which in turn depends on environmental conditions. Notwithstanding the difficulty of considering a phage an organism, it not only can reproduce itself (albeit hijacking the cellular components and machinery) but also responds to environmental conditions. For example, the λ phage may prefer lysogenic to lytic growth under conditions of starvation, perhaps since starving cells cannot provide components supporting efficient lytic growth [110]. Starving bacterial cells produce low amounts of proteases, which, at high concentrations observed in rich medium, destroy the phage CII protein required for the activation of the phage *cI* and *int* genes essential for establishing lysogeny. The CI repressor produced under conditions of high CII activity inhibits transcription from the divergent pR and pL promoters in the λ regulatory region and thus turns off the expression of all the phage genes except that of its own. However, if CII is rapidly degraded, no CI repressor is synthesized, the Cro repressor occupies the λ regulatory region instead of CI, and lytic growth ensues. So, while the phage senses the energy status of the cell, this latter is ultimately translated into specific nucleoprotein complexes competing for binding at the λ regulatory region and acting as a switch between alternative

developmental pathways. Furthermore, the regulation of the lysogenic/lytic switch by CI repressor appears sensitive to DNA supercoiling [112,113], as is the RNAP σ70/σS holoenzyme switch in *E. coli* [36]. A similar relationship of DNA topology-dependent competitive binding at the overlapping DNA sites has been observed between the early and late NAPs, FIS and Lrp, respectively, involved in the control of the type 1 fimbrial genetic switch in *E. coli* [122].

Ptashne [110] suggested that the regulatory sequences initiated at the λ control region essentially generate a ‘cascade’ along each pathway, sequentially turning *on* and *off* groups of genes. In this cascade, one regulatory protein turns *on* or *off* a block of genes, which includes another regulatory gene, the product of which in turn regulates another block of genes and so on. Here, the regulatory cascade is established by protein binding only at a few DNA sites on the phage genome. This type of regulation is made possible due to the peculiar spatial organization of functionally related genes in the genome, as they are grouped together and also transcribed in the same direction. Thus, while emphasizing the role of cascades, this mode of regulation also implicates the spatial gene organization and the directional extension of transcription—properties considered here as belonging to the analog (continuous) information type—in contrast to the ‘cascade control’, which essentially turns the genes *on* or *off* and therefore provides purely digital information.

Despite the differences in complexity and details, in both the bacterial and phage systems, there is a discernible common organizational design: initial utilization of analog information (spatial *oriC-ter* gradients of DNA binding sites interacting with temporal gradients of regulatory proteins in the former, and gradually extending transcription starting from the divergent pR and pL promoters in the latter) and its subsequent conversion into digital information (the differing nucleoprotein complexes producing specific gene expression patterns sustaining either reproduction or maintenance in the former, and the distinct sets of regulatory proteins underpinning either the lytic or lysogenic pathway in the latter).

## 5 Analog/Digital Information Conversion Operates as a Regulatory Device in Living Systems of Diverse Structural Complexity

Given the similarity of the underlying regulatory design in the bacterial and phage systems, the pertinent question is whether this mode of information conversion occurs also in more complex multicellular organisms. Indeed, over three decades ago, Ptashne [110] drew parallels between the processes of gene regulation in phage λ and higher organisms, in particular the process of *Drosophila* embryogenesis, where the formation of the pattern of stripes expressing the segmentation gene even-skipped (*eve*) depends on the sequential turning on and off of transcriptional regulators, a form of cascade control similar to that of the λ life cycle. Actually, during *Drosophila* embryogenesis, notwithstanding the role of the digital on or off type ‘cascade control’, the importance of analog information in the pattern formation is most conspicuous.

**Figure 9:**
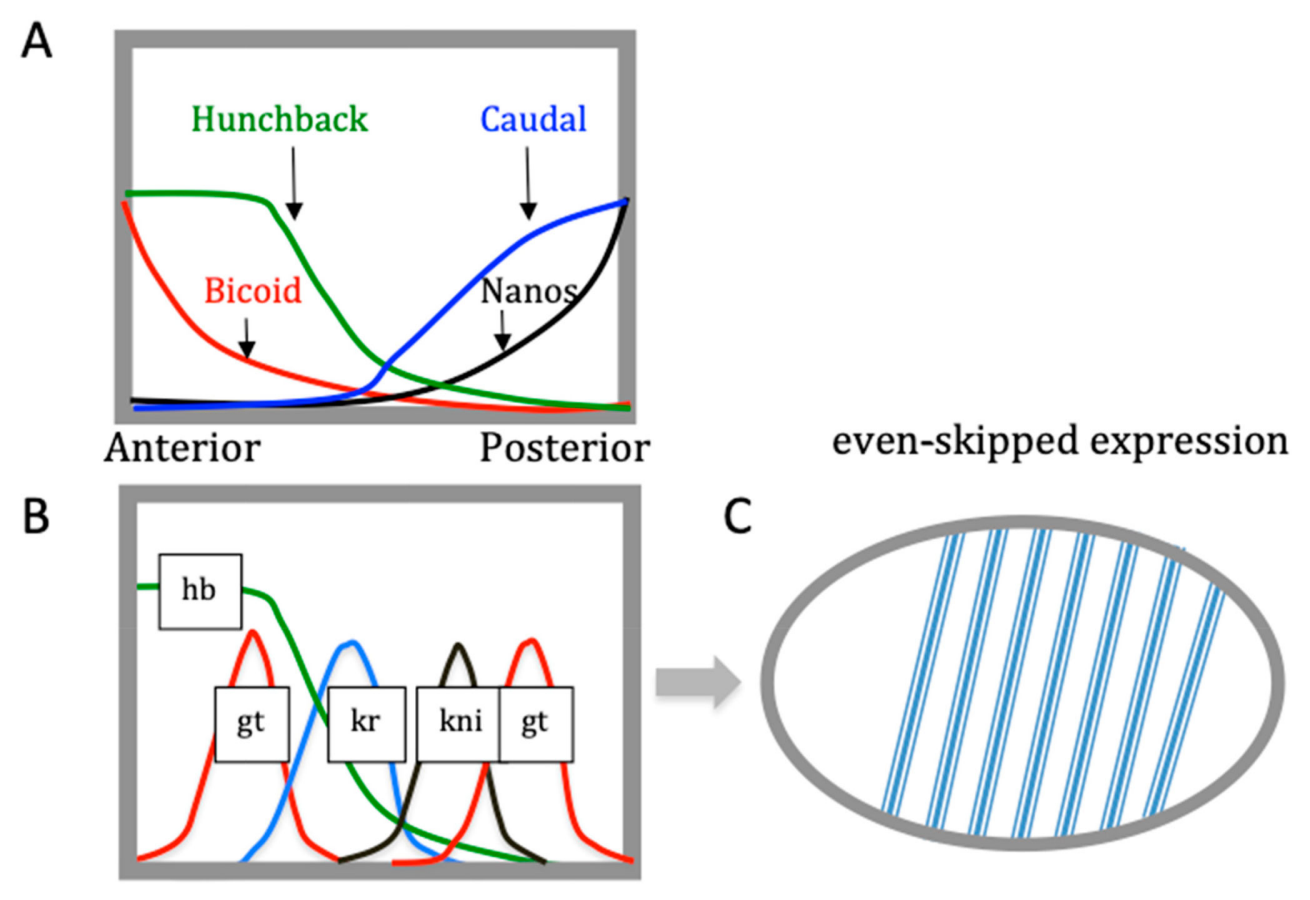
Generation of the anterior−posterior pattern of even-skipped (*eve* pair-rule gene) expression initiated by gradients of the *Drosophila* maternal effect genes. (**A**) The Bicoid protein gradient extends from anterior to posterior, while the Nanos protein gradient extends from posterior to anterior. Nanos inhibits the translation of the *hunchback* message (in the posterior), while Bicoid prevents the translation of the *caudal* message (in the anterior). This inhibition results in opposing Caudal and Hunchback gradients. (**B**) Spatial distribution of Bicoid-responsive segmentation (gap) gene expression such as giant (gt), krueppel (kr), and knirps (kni). The gap gene products mutually repress each other’s expression, resulting in spatially defined domains of gap gene expression along the anterior−posterior axis of the embryo, which ultimately define the pattern of even-skipped (a pair-rule gene) expression in seven stripes (**C**). Each stripe has an independent transcriptional control system consisting of different constellations of transcription factors (specific combinations of activators) interacting with seven different enhancer sequences, all of which can activate even-skipped gene expression.

During *Drosophila* embryonic development, the maternal gene messages are strategically deposited at opposite—anterior and posterior—poles of the embryo, such that the translated proteins diffuse from the poles forming spatial concentration gradients along the anterior−posterior axis (Figure 9). These overlapping concentration gradients lead to spatially determined, locally fixed ratios of transcriptional regulators and thus establish boundaries of target gene expression. The spatially determined threshold concentrations of transcriptional regulators lead to the sequential activation of the various segmentation genes, eventually producing a distinct pattern of seven stripes expressing the even-skipped pair-rule gene, which is essential for the segmentation of the embryo [123,124]. So, here we have a clear case of the conversion of analog information (continuous protein concentration gradients) into digital information (specific pattern of seven discrete *eve* stripes). This conversion of protein concentration gradients into a particular pattern of stripes is enabled by the existence of seven distinct enhancers of the *eve* gene (one for each stripe), each of which binds different combinations of regulatory proteins depending on their spatially determined threshold concentrations established along the anterior−posterior axis. Thus, seven distinct enhancers binding different combinations of regulatory proteins generate alternative nucleoprotein complexes independently activating *eve* expression—albeit in a spatially defined manner—and producing the specific pattern of seven stripes.

At this stage of development (syncytial blastoderm), the *Drosophila* embryo is not yet cellularized and contains about 1500 nuclei evenly distributed underneath the membrane, while each stripe extends over six nuclei on average [110]. The *Drosophila* genome is about 180 Mb in size, so a single row of nuclei expressing even-skipped gene would contain about 1 Gb of DNA. In contrast, the *E. coli* genome is 4.6 Mb in size, and that of phage λ is about 48.5 Kb. The *Drosophila* embryo is (longitudinally) about 500 times the size of an *E. coli* cell. Thus, concerning the spatial extension of implicated gradients, there is a difference in orders of magnitude. Furthermore, in the case of phage λ and *E. coli*, the gradients (directionally elongating transcripts in the former and the putative sigma factor and NAP gradients in the latter) extend over single genomes (albeit differing in size by two orders of magnitude), whereas in the case of *Drosophila*, the protein concentration gradients extend over more than a thousand spatially arranged genomes (nuclei).

Finally, in the phage and bacterial genomes, the regulatory proteins have relatively easy access to DNA binding sites, whereas the binding of cognate regulatory sites is obstructed in *Drosophila* nuclei by the tight packaging of the DNA in chromatin. Furthermore, in phage λ, the spatial extension of genomic transcription produces distinct sets of proteins that are put to work in temporal succession. The temporal gradients of NAPs and sigma factors do not coexist in a single bacterial cell but are successively established in the progeny. In contrast, the opposite concentration gradients of Bicoid and Nanos extending from the poles and responsible for the formation of the anterior and posterior structures coexist in a single *Drosophila* embryo.

Notwithstanding the abovementioned differences, there are also remarkable similarities between these systems. During *Drosophila* embryonic development, as well as during the *E. coli* growth cycle, the transcriptional and metabolic programs appear tightly correlated [56,125,126]. The regulation of the activity of DNA topoisomerases is associated with both the *E. coli* growth cycle and *Drosophila* embryogenesis [27,127]. Additionally, the *Drosophila* development time, the phase transition during the *E. coli* growth cycle, and the λ phage lytic/lysogeny decision all respond to nutritional supply [110,128,129]. However, the most important similarity between the systems is the phenomenon of the conversion of continuous data (analog information) into discrete data (digital information). First, in all three systems, there is a directional dispersion of analog information (gradients of proteins in *Drosophila* and *E. coli* and continually elongating transcripts in phage λ) from spatially localized sources (anterior and posterior poles in *Drosophila*, proximities of the chromosomal *oriC* and *ter* poles in *E. coli*, and divergent pR and pL promoters in the regulatory region of phage λ). Second, in all three systems, the conversion of analog into digital information is manifest in the formation of distinct nucleoprotein complexes involving DNA binding proteins interacting with DNA sites spatially organized in corresponding genomes or in the embryo. In the latter case, this interaction is facilitated and fine-tuned by ATP-driven chromatin remodelers [130]. Finally, and more generally, assuming that the initial gradients possess higher entropy than the ensuing discrete patterns of DNA-protein interactions, we may also assume an energy-driven decrease in entropy associated with the pattern-making in all three systems.

**Figure 10:**
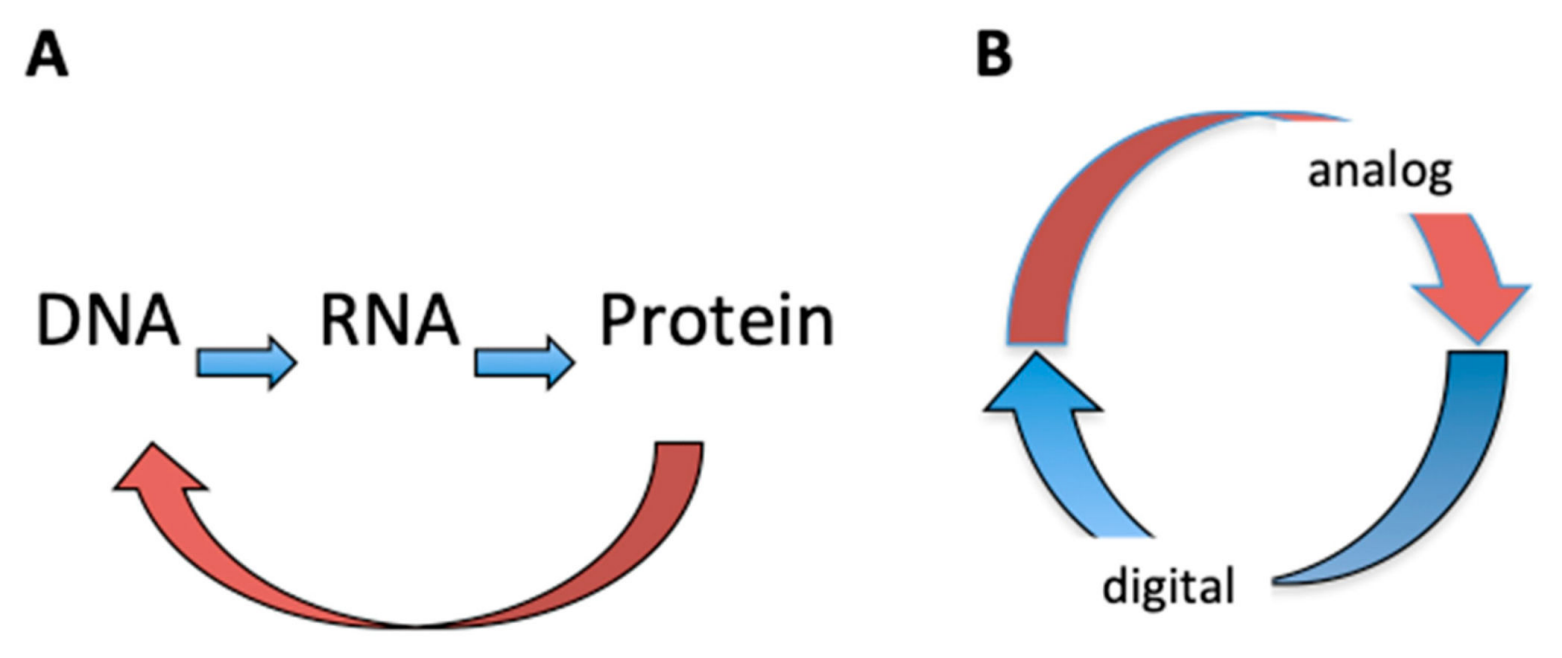
Organization of information flow and inter-conversion in the living system. (**A**) Depicted is the central dogma of molecular biology: digital genetic information flows only in one direction, from DNA, to RNA, to protein (blue arrows). However, the DNA analog information is recognized directly by the DNA binding proteins stabilizing various 3D conformations of the DNA (curved brown arrow) and thus modulating the content of digital information. (**B**) Systems-theoretical model for the interconversion of DNA information. The terms ‘analog’ and ‘digital’ refer to two distinct information types stored in the genomic DNA polymer. DNA analog information implies both static (sequence organization viz. arrangement of base steps) and dynamic parameters (DNA 3D configuration and superhelicity). Digital information implies the differential gene expression patterns and the gene interaction networks (including regulatory cascades) emerging thereof. Analog information provides an integrative sensory interface for both internal and external signals as well as a regulatory context for digital code expression, whereas the latter provides information for reproduction and the maintenance of the former. Together, the inter-converting DNA ‘codes’ form a coordinated self-referential circuit responding to both the internal and external signals as an indivisible unity.

Thus, despite the substantial differences in size, complexity, and structural detail between these three living systems (although the phage system can barely qualify as such), in all cases, we have a spatiotemporally organized gene regulation program. The temporally organized regulatory cascades alone cannot provide for the unity of the living system—a cascade has a beginning and an end—yet it does not necessarily close onto itself, while as mentioned above, from the systems-theoretical perspective, the living system constitutes a self-referential circuit. What is assumed here is the closure of the system onto itself, and this organization of unity implicates spatial coordinates [131–133]. A relevant example of the integration of cell division and differentiation by coordinating the temporal gene expression and spatial organization of gene products and protein gradients has been provided in studies of the *Caulobacter crescentus* system [9,134,135]. All three systems discussed here have a similar organization embodied in the conversion of two distinct information types manifesting a coordinated unity (Figure 10). In this view, a phage acquires the properties of a living system primarily by tapping into its intrinsic organization, namely, appropriating the device of analog/digital information conversion.

## 6 Conclusions

The central dogma of molecular biology states that genetic information flows only in one direction, from DNA, to RNA, to protein, or from RNA directly to protein. This theory, highlighting the unidirectional flow of genetic information, does not consider the DNA analog information and the crosstalk between the DNA and DNA binding proteins—essentially a feedback loop (Figure 10A, brown curved arrow). It thus cannot account for the main organizational hallmark of living systems manifesting a self-referential circuit. We argue here that this latter organizational feature is inherent in the structure of the DNA double helix, representing a basic device for interconverting information. This conversion of information is made possible by the existence of two logically different—digital and analog—information types stored in the DNA. While the digital genetic information encodes all the DNA binding proteins and enzymes, the DNA appears to ‘read itself’ via DNA−protein interactions. These interactions are informative as they lead to the modulation of gene expression according to nascent external and/or internal signals. The coordinated DNA ‘self-readout’ mediated by DNA binding proteins is based on a strategic spatial organization of regulatory DNA binding sites and regulated genes in the genome (or the spatial organization of nuclei in the case of *Drosophila* embryos). This spatial organization in turn is determinative for the successive formation of distinct regulatory nucleoprotein complexes and the emergence of temporal regulatory ‘cascades.’ Thus, the DNA genome can generate spatiotemporally coordinated patterns of activated and repressed genes in response to both internal and external signals. We infer that the genomic DNA, acting as a double-coding device, represents the integrative interface where ‘that which monitors and that which is monitored’ meet to generate a coordinated self-referential unity (Figure 10B) responding to both the internal and external signals as an indivisible whole.

Finally, we note that the concept of information conversion emphasizes the importance of therapeutic approaches focused on the development of novel drugs targeting global regulators such as NAPs and DNA topoisomerases [136–138], as well as particular sequences and/or structural features of the DNA [139–141]. Research along these lines may eventually pave the way to the discovery of new approaches for modulating the developmental plasticity—the capacity of the genotype to produce different phenotypes—and thus restricting high phenotypic variation and the potential of the emergence of abnormal developmental trajectories [142,143]. This approach may also prove useful for the assessment of the relative contribution of genes and the environment to the development of disease [144,145] as well as for the understanding of the evolution of gene regulation [146].

## Figures and Tables

**Table 1:** Free stacking/melting energy of the ten DNA base steps [14].

Base Steps	kcal/mol
AA/TT	−1.02
AT/AT	−0.73
TA/TA	−0.60
CA/TG	−1.38
GT/AC	−1.43
CT/AG	−1.16
GA/CT	−1.46
CG/CG	−2.09
GC/GC	−2.28
GG/CC	−1.77

**Table 2:** Estimated number and concentrations of the most abundant nucleoid-associated proteins in *E. coli* [121]. Approximate numbers for RNA polymerase and *lac* repressor are shown for comparison.

Protein	Exponential Phase		Early Stationary Phase	
	No./Cell	Concn (μM)	No./Cell	Concn (μM)
Dps	8000	7	120,000	100
FIS	60,000	50	Not detectable	
H-NS	20,000	17	15,000	13
HU	55,000	45	25,000	20
IHF	10,000	8	50,000	41
StpA	25,000	28	15,000	17
Totals		155		191
LacI (LacR)	10			
NAP	4000−6000			

## Data Availability

No new data were created in this study.
